# Concordance and reproducibility in the location of reference points for a volumetric craniofacial analysis: Cross-sectional study

**DOI:** 10.34172/joddd.2023.37025

**Published:** 2023-07-17

**Authors:** Natali Romero-Tapiero, Andrés Giraldo-Mejía, Adriana Herrera-Rubio, Juan Fernando Aristizábal-Pérez

**Affiliations:** ^1^Department of Orthodontics, Faculty of Health, Universidad del Valle, Cali, Colombia; ^2^Department of Orthodontics, Faculty of Health, Universidad CES, Medellín, Colombia

**Keywords:** Cone-beam computed tomography, Three-dimensional image, Orthodontics, Cephalometry

## Abstract

**Background.:**

Considering the limitations of visualization that occur even with the use of radiographs, the cone beam computed tomography (CBCT) becomes more attractive to diagnose and propose an assertive treatment plan. This study aimed to evaluate intra and interobserver reproducibility, and concordance of 31 reference points we described considering visualization tools and the three planes of space in a bimaxillary CBCT.

**Methods.:**

Three observers located in triplicate the 31 reference points in the CBCT of six healthy patients. Friedman test was used to compare intraobserver paired samples, and interobserver concordance was determined by the intraclass correlation coefficient (ICC) with ranges>0.75 (excellent), between 0.60 and 0.74 (good), between 0.40 and 0.59 (sufficient) and<0.40 (poor). The *P* value was set at<0.05.

**Results.:**

A high ICC (>0.75%) was obtained by comparing the x, y, and z values at the location of landmark points. Excellent ICC>0.75 was for 81.7% and poor<0.40 was 7.5% in the interobserver evaluation. Data showed that 25 points had excellent concordance on the x-plane, 25 on the y-plane, and 26 on the z-plane (0.75%).

**Conclusion.:**

Intraobserver concordance analysis indicated that location of anatomical reference points on bimaxillary CBCT is performed with great reproducibility by interpreting their location with a clear description in the three planes of space. Complexity of achieving a good precision degree in the manual marking of reference points caused by convexities of the anatomical structures involved, might explain the variability found. The systematized location of the reference points would contribute to reduce such variability.

## Introduction

 Cephalometric analysis is the preferred diagnostic study in the orthodontic area.^1-3^ Over the years, it has been implemented to identify the cause of malocclusion. Due to its application in orthodontics, radiographic evaluation should create a representation that is as close to the reality of the patient as possible, allowing the clinician to understand the causes of the existing craniofacial alterations and the alternatives for their correction.^1,4^ This diagnostic practice has been based mainly on the study of lateral skull radiographs, but other cranial radiographs are added to the diagnostic process.^5^ On the other hand, taking into account the limitations of visualization that occur even with the use of radiographs, the cone beam computed tomography (CBCT) becomes more attractive to make the diagnosis, and consequently an assertive treatment plan.^3^ Cephalometric analysis is designed to be performed on a 2D image,^6^ so conventional cephalometric analysis tends to be performed on images that have three-dimensional (3D) information, and the additional information contained in a CBCT is not used.

 CBCT represents a radiation exposure that could be of higher dose,^6^ however, this depends on the quality of the equipment and the settings by the manufacturers or by adjusting the image parameters that the CBCT machine allows manually. The acquisition of three-dimensional images with very low radiation doses and sufficient quality for diagnosis has already been reported.^7–9^

 In this area, some studies describe the marking of reference points in the CBCT and make the respective comparison of reliability and reproducibility between the two-dimensional and three-dimensional methods,^10^ which yield favorable data for the three-dimensional analysis. However, there is a need to adjust the description of volumetric points for a three-dimensional image and make a diagnostic tool.^11^ The description of the points in the planes, x, y is already done and now z is necessary to be describe. It has been described in the literature that the points that present great difficulty and variability in their identification in the three planes of space are some such as the porion, condilion, orbital, basion, gonion, anterior nasal spine, posterior nasal spine, and the apex of the lower incisor.^12^

###  Justification

 Visualization tools (zoom and contrast) are useful for marking reference points with high reliability.^13^ A step forward is taken, because the technology seeks to facilitate and simplify these processes.^6^ Due to the information obtained from the CBCT, it is necessary to develop methods that allow its use through the standardization of cephalometric measurements in a 3D reconstruction. The objective of this study was to indicate 31 reference points describing them in the three planes of space in a bimaxillary CBCT and to evaluate the intra and interobserver reproducibility and concordance.

## Methods

 CBCT of patients who attended the [Universidad del Valle, Cali, Colombia] dental school seeking orthodontic treatment were selected. The tomography of patients with complete permanent dentition and in which it was visualized from the nasion point to the menton point was included. CBCT of patients with skeletal abnormalities, syndromes, with a history of craniofacial trauma, and a history of orthognathic surgery were excluded.

 Using the following formula:


n=2Zα2+Zβ2s2e2

We determined the size of the adequate sample to detect a difference > 0.5 mm between the reference points.^3^ From a database of 108 bimaxillary CT scans taken between June 2019 and March 2020, 20 met the selection criteria, using a simple random sampling the six tomographies were selected.

 The images were generated by an iCAT 1719 equipment, FOV 12.6 cm, voxel size 0.25. resolution 0.25 voxel per 26.9 sec exposure + 37.07 kVp 120 and size of the reconstructed volume diameter of 16 cm. The images were taken with the patients adopting a corrected posture with a straight back and head at rest, with the Frankfort plane parallel to the floor.

**Figure 1:**
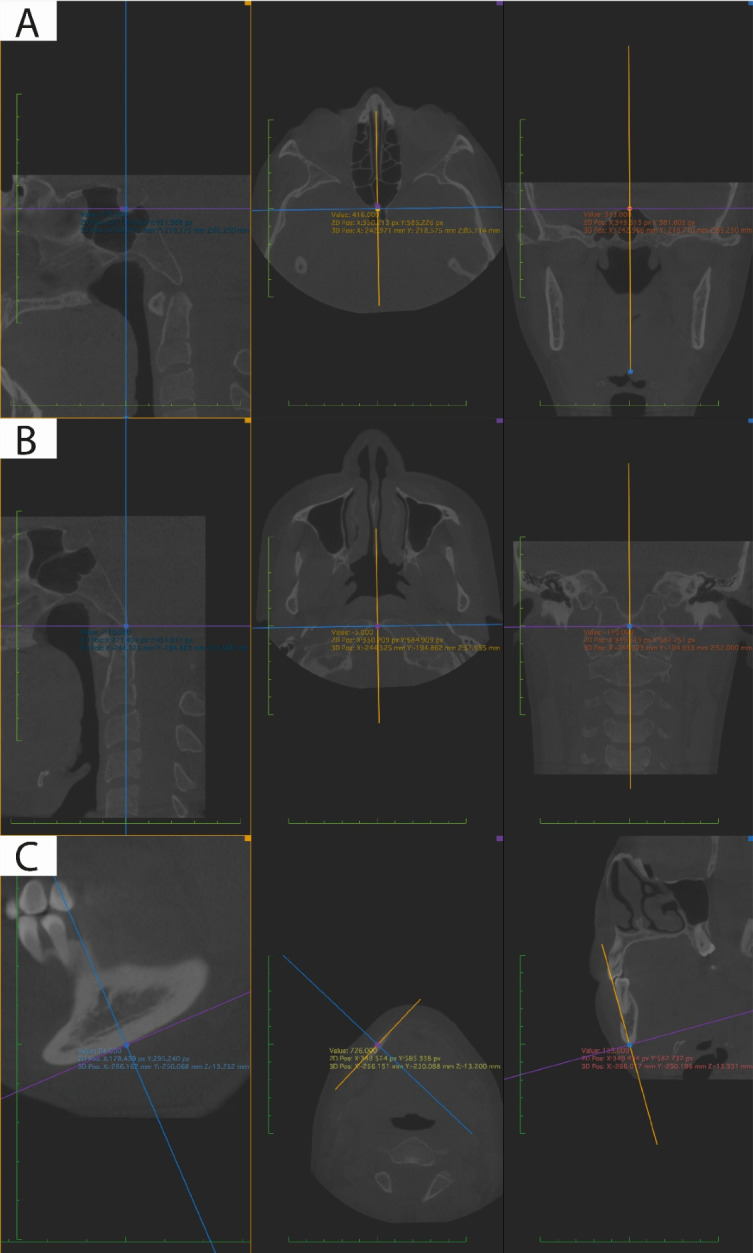
Reference points marked in the multiplanar view taking into consideration the three planes of space (sagittal, axial, and coronal). A. Sella point, B. Basion point, C. Mandibular base point

 Tomographies were exported in DICOM files for viewing in Horos software (version 4.0.0; The Horos Project & OsiriX Team) in multiplanar 3D mode (MPR). The marking of each point was verified three dimensionally (Tables 1-3). The point was represented in the three windows of the spatial planes x, y, and z, each observer collected the information on the coordinates of the reference points employing image captures to record the information in tables of Microsoft Excel for Mac version 16.52 (Figure 1).

**Table 1:** Skull base reference points. The reference points are presented by segments, skull base, maxilla, and mandible

**Name of RP**	**RP**	**Coronal**	**Sagittal**	**Axial**
Sella	S	***Verify that the sagittal axis passes through the midline of the cranial structure from right to left. The point is marked on the floor of the sella.	**On the midsagittal plane. Locate the deepest point in the concavity of the floor of the sella turcica.	*Verify that the sagittal axis passes through the midline of the cranial structure in an anteroposterior direction.
Nasion	Na	***Verify that the sagittal axis passes through the midline of the cranial structure. Mark the point on the fronto-nasal suture and coincident with the midline.	**On the midsagittal plane, mark the most anterior point of the junction of the nasal bones with the frontal.	*Verify that the sagittal axis passes through the midline of the cranial structure. Position the point on the anterior border of the fronto-nasal suture.
Right Fossa	Fr	***Locate the sagittal axis on the longitudinal axis of the right mandibular ramus. Locate the point at the top of the concavity of the right glenoid fossa.	*From the axial view, locate the sagittal section at the level of the right mandibular ramus. From the sagittal view locate the coronal axis on the axis of the right condyle, and the axial axis over the head of the right condyle. Mark the point at the top of the concavity of the right glenoid fossa.	**Locate the coronal axis along the lateral and medial poles of the right condyle.
Left Fossa	Fl	***Locate the sagittal axis on the longitudinal axis of the left mandibular ramus. Locate the point in the uppermost part of the concavity of the left glenoid fossa.	*From the axial view locate the sagittal section at the level of the left mandibular ramus. From the sagittal view locate the coronal axis on the axis of the left condyle, and the axial axis over the head of the left condyle. Mark the point at the top of the concavity of the left glenoid fossa.	**Locate the coronal axis along the lateral and medial poles of the left condyle.
Basion	Ba	***On the midsagittal plane mark the most inferior and central point of the basilar process.	**On the midsagittal plane, mark the point at the lowest part of the basilar process.	*Locate the sagittal axis on the midline. Mark the point on the edge of the basilar process coinciding with the axial axis of the odontoid.
Right fronto-malar suture	Ft-r	*On the axial view, move the axis of the coronal plane anteriorly until you see the right fronto-malar suture. Then move the sagittal axis to the most lateral part of the right fronto-malar suture. Mark the point at the extreme lateral part of the right fronto-malar suture.	**Mark the point at the most anterior part of the right fronto-malar suture.	***Mark the point on the most anterior and lateral part of the right fronto-malar suture.
Left fronto-malar suture	Ft-l	*On the axial view, move the axis of the coronal plane anteriorly until the left fronto-malar suture is visualized. Then move the sagittal axis to the most lateral part of the left fronto-malar suture. Mark the point at the extreme lateral part of the left fronto-malar suture.	**Mark the point at the most anterior part of the left fronto-malar suture.	***Mark the point on the most anterior and lateral part of the left fronto-malar suture.

* First plane to locate. **Second plane to locate. ***Third plane to locate. RP: reference points. The signaling of the description of the reference points has been used to indicate in which order the location of these is carried out.

**Table 2:** Reference points in the maxilla

**Name of RP**	**Coronal**	**Sagittal**	**Axial**
ENA	***Verify this point with the axial and sagittal planes.	** Mark the point on the anterior part of the anterior nasal spine.	*Locate the sagittal axis on the midline Mark the point on the anterior part of the anterior nasal spine coinciding with the midline.
ENP	***Verify this point with the axial and sagittal planes.	**Mark the Point on the back of the posterior nasal spine.	*Locate the sagittal axis on the midline. Mark the point on the back of the posterior nasal spine coinciding with the midline.
Mx 6 R	*Displace the axial axis at the level of the maxillary right molars. Verify that it is the outermost point on the maxillary cortex.	***Mark the point at the apex of the furcation of tooth 16.	**Displace the coronal axis on tooth 16 following a central axis in the vestibular-palatal direction. Verify that it is the outermost point on the maxillary cortex.
Mx 6 L	*Displace the axial axis at the level of the maxillary left molars. Verify that it is the outermost point on the maxillary cortex.	***Mark the point at the apex of the furcation of tooth 26.	**Displace the coronal axis on tooth 26 along a central axis in the vestibular–palatal direction. Verify that it is the outermost point on the maxillary cortex.
Mx 3 R	***The point must be marked in the most anterior part of the contour of tooth 13	**From the sagittal view you should see the Mx 6 R point. Displace the axial axis parallel to the occlusal plane and aligned it with the Mx 6 R point so that the coronal axis is located over tooth 13. The point must be marked in the most anterior part of the contour of tooth 13	*On the axial view where you see the Mx 6 R point, move the coronal axis towards tooth 13 without losing sight of the Mx 6 R point.
Mx 3 L	***The point must be marked in the most anterior part of the contour of tooth 23	**From the sagittal view you should see the Mx 6 I point. Displace the axial axis parallel to the occlusal plane and aligned it with the Mx 6 I point so that the coronal axis is located over tooth 23. The point must be marked in the most anterior part of the contour of tooth 23	*On the axial view where you see the Mx 6 L point, move the coronal axis towards tooth 23 without losing sight of the Mx 6 L point. The point must be marked in the most anterior part of the contour of tooth 23, with the sagittal plane aligned with Mx 6 L.
Tuberosity R	*Displace the axial axis at the level of the maxillary right molars. Also, displace the axial axis to visualize the junction between the pterygoid and the maxilla on the axial view. Mark the point laterally on the right pterygoid cortex.	***Displace the axial axis caudally until reaching the most inferior point of the pterygopalatine fossa. Mark the lowest point at the junction of the pterygoid and maxilla.	**Displace the coronal axis with the pterygopalatine fossa. Mark the point laterally on the right pterygoid cortex.
Tuberosity L	*Displace the axial axis at the level of the maxillary left molars. Also, pan the axial axis to visualize the junction between the pterygoid and the maxilla on the axial view. Mark the point laterally on the left pterygoid cortex.	***Displace the axial axis caudally until reaching the most inferior point of the pterygopalatine fossa. Mark the lowest point at the junction of the pterygoid and maxilla.	**Displace the coronal axis with the pterygopalatine fossa. Mark the point laterally on the left pterygoid cortex.
Infraorbital R	**Displace the axial axis and sagittal axis with the medial zone of the infraorbital foramen. Mark the point at the top of the right infraorbital foramen.	***In this view you should see the anterior portion of the infraorbital canal. Mark the point on the most superior and anterior portion of the right infraorbital foramen.	*Displace the coronal axis anteriorly until the right infraorbital foramen is visible in the coronal view. Mark the point on the most anterior portion of the cortical bone surrounding the infraorbital foramen.
Infraorbital L	**Displace the axial axis and sagittal axis with the medial zone of the infraorbital foramen. Mark the point at the top of the left infraorbital foramen.	***In this view you should see the anterior portion of the infraorbital canal. Mark the point on the most superior and anterior portion of the left infraorbital foramen.	*Displace the coronal axis anteriorly until the left infraorbital foramen is visible in the coronal view. Mark the point on the most anterior portion of the cortical bone surrounding the infraorbital foramen.

RP: reference points * First plane to locate. **Second plane to locate. ***Third plane to locate. RP: reference points.

**Table 3:** Reference points in the mandible

**Name of RP**	**Coronal**	**Sagittal**	**Axial**
6 Inf R	*Desplace el eje axial a nivel de los molares mandibulares derechos.	***Locate the point at the top of the convexity of the 46 furcation.	**Locate the sagittal axis on the tooth line of the mandibular right molars, and the coronal axis on the vestibulolingual central axis of tooth 46. Mark the point on the inner aspect of the vestibular cortex of tooth 46.
6 Inf L	*Displace the axial axis at the level of the mandibular left molars.	***Locate the point at the top of the furcation convexity of 36.	**Locate the sagittal axis on the tooth line of the mandibular left molars, and the coronal axis on the vestibulolingual central axis of tooth 36. Mark the point on the inner aspect of the vestibular cortex of tooth 36.
3 inf R	***The point must be marked in the most anterior part of the contour of tooth 43	**From the sagittal view you should see the 6 inf R point. Displace the axial axis parallel to the occlusal plane and aligned it with the 6 inf R point in such a way so that the coronal axis is located on tooth 43. The point must be marked in the most anterior part of the contour of tooth 43	*On the axial view where the 6Inf R point is visualized, move the coronal axis towards tooth 43 without losing sight of the 6 inf R point. The point must be marked in the most anterior part of the contour of tooth 43, with the sagittal plane aligned with 6 inf R.
3 inf L	***The point must be marked in the most anterior part of the contour of tooth 43	**From the sagittal view you should see the 6 inf L point. Move the axial axis paralle23 l to the occlusal plane and aligned with the 6 inf L point in such a way so that the coronal axis is located on tooth 33. The point must be marked in the most anterior part of the contour of tooth 33	*On the axial view where the 6Inf L point is visualized, move the coronal axis towards tooth 33 without losing sight of the 6 inf L point. The point must be marked in the most anterior part of the contour of tooth 33, with the sagittal plane aligned with 6 in L.
Condylium R	***Displace the sagittal axis along the axis of the condyle, and also displace the axial axis to the head of the condyle. Locate the highest point on the convexity of the head of the right condyle.	**Displace the coronal axis along the axis of the condyle. Locate the highest point of the convexity of the head of the right condyle.	*Displace the sagittal axis to the right side of the mandibular condyle area. Rotate the coronal axis Following the axis of the medial lateral condyle. The point will be visualized in the middle of the head of the condyle.
Condylium L	***Displace the sagittal axis along the axis of the condyle. Also, move the axial axis to the head of the condyle. Locate the highest point on the convexity of the head of the left condyle.	**Displace the coronal axis along the axis of the condyle. Locate the highest point of the convexity of the head of the left condyle.	*Displace the sagittal axis to the left side of the mandibular condyle area. Rotate the coronal axis Following the axis of the medial lateral condyle. The point will be visualized in the middle of the head of the condyle.
Gonion R	*Displace the axial axis at the level of the mandibular body. Verify that the point is on the lowest portion of the right mandibular ramus.	***Rotate the sagittal axis along the axis of the mandibular condyle and ramus. The point is located at the midpoint of the mandibular angle between the ramus and the mandibular body.	**Locate the sagittal axis along the mandibular ramus. The point should be located on the posterior border of the mandibular body.
Gonion L	*Displace the axial axis at the level of the mandibular body. Verify that the point is on the lowest portion of the left mandibular ramus.	***Rotate the sagittal axis along the axis of the mandibular condyle and ramus. The point of intersection is the midpoint of the left mandibular angle.	**Locate the sagittal axis along the mandibular ramus. Verify that the point is on the most posterior portion of the left mandibular body.
Base Mad R	**Point coinciding with the exit of the mental foramen on the lower edge of the mandibular body on the right side.	*Following the axis of the mandibular body, at the level of the posterior wall of the right mental foramen, the coincident point on the edge of the mandibular body.	***Verify that the point is located on the outermost cortical of the right mandibular body.
Base Mad L	**Point coinciding with the exit of the mental foramen on the lower edge of the mandibular body on the left side.	*Following the axis of the mandibular body, at the level of the posterior wall of the left mental foramen, the coincident point on the edge of the mandibular body.	***Verify that the point is located on the outer cortical bone of the left mandibular body.
Pogonion	***This view shows the pogonion projection with the midline facial structures. It does not necessarily have to be aligned with ENA or N.	*Displace the coronal axis with the most anterior point of the mandibular symphysis.	**Displace the coronal and sagittal axis to the most anterior point of the mandibular symphysis.
Menton	**Displace the axial and coronal axis to the lowest point of the menton contour.	*Displace the axial and coronal axis to the lowest point of the menton contour. Inferior point of the mandibular symphysis.	***Displace the sagittal and coronal axis to the most anterior point of the chin contour.
Coronoid R	*Displace the sagittal axis to the far right until the coronoid process is visible in the sagittal view. Most superior point of the right mandibular process.	**Displace the axial axis with the superior portion of the right mandibular process. Most superior and anterior point of the right mandibular process	***Most anterior point of the right mandibular process.
Coronoid L	*Displace the sagittal axis to the far right until the coronoid process is visible in the sagittal view. Most superior point of the left mandibular process.	**Displace the axial axis with the superior portion of the left mandibular process. Most superior and anterior point of the left mandibular process.	***Most anterior point of the left mandibular process.

RP: reference points. * First plane to locate. **Second plane to locate. ***Third plane to locate. RP: reference points.

 Three observers were involved, two of them were orthodontists, and one was a dental radiologist. 31 reference points were chosen, and these were reviewed in two meetings in which pilot tests were carried out to describe them three-dimensionally, considering anatomical and technological criteria. A consensus was sought among the three observers that the landmarks were clearly described. These 31 reference points were marked in the multiplanar view in triplicate by each of the observers individually.

###  Statistical analysis

 The Friedman test was used to make a comparison of paired or related intraobserver samples by marking the 31 reference points in their three coordinates *x*, *y*, and *z* in triplicate. Interobserver concordance was determined by the intraclass correlation coefficient (ICC) with the ranges > 0.75 (excellent), between 0.60 and 0.74 (good), between 0.40 and 0.59 (sufficient), and < 0.40 (poor). The *P* value to determine statistical significance for differences between coordinates was set at < 0.05 and the difference in millimeters of the reference points found with poor concordance was obtained.

## Results

 The evaluation of the points in the 3 planes of space *x, y,* and *z* was carried out using the Friedman test to identify if these differences were statistically significant, with which no significant differences were found (*P* > 0.05) for 19 landmarks (Table 4).

**Table 4:** Intraobserver reliability data evaluated with Friedman’s analysis *P value ( < 0.05)

**Reference point**	**Friedman**								
	**Observer 1**			**Observer 2**			**Observer 3**		
	**x**	**y**	**z**	**x**	**y**	**z**	**x**	**y**	**z**
Sella	0.311	0.846	0.538	0.513	0.115	0.348	0.846	0.311	0.58
Nasion	0.311	0.846	0.368	0.311	0.607	0.186	0.135	0.846	0.717
Right Fossa	0.311	0.011*	0.092	0.311	0.03*	0.861	0.135	0.846	0.247
Left Fossa	0.607	0.223	0.229	0.311	0.223	1	0.607	0.607	0.031*
Basion	0.115	0.513	0.549	0.607	0.135	0.678	1	0.607	0.065
Right fronto-malar suture	0.513	0.846	0.28	0.311	0.607	0.467	0.513	0.069	0.26
Left fronto-malar suture	0.846	0.311	0.076	0.846	1	0.305	0.607	0.311	0.957
ENA	0.607	0.607	0.513	0.069	0.311	0.247	0.607	0.846	0.28
ENP	0.311	0.311	8.67	1	0.607	0.738	0.135	0.513	0.264
6 Inf R	0.115	0.115	0.036*	0.846	0.311	0.115	0.513	0.607	0.676
6 Inf L	0.223	0.846	0.873	0.846	0.607	1	0.115	0.513	0.705
3 inf R	0.311	0.846	1	0.311	1	0.607	0.846	0.115	0.846
3 inf L	0.846	0.223	0.846	1	0.069	0.115	0.135	0.607	0.846
Mx 6 R	0.846	0.311	0.196	0.513	0.846	0.846	0.183	0.607	0.738
Mx 6 L	0.311	0.115	0.368	0.846	0.03*	0.115	0.607	0.607	0.846
Mx 3 R	0.311	0.011*	0.115	0.513	0.513	0.513	0.311	0.03*	0.115
Mx 3 L	0.513	0.607	0.223	0.311	0.311	0.513	0.311	0.311	0.957
Tuberosity R	0.223	0.135	0.878	0.042*	0.311	0.311	0.846	0.846	0.223
Tuberosity L	0.607	0.311	0.186	0.223	0.042*	0.513	0.607	0.607	0.727
Infraorbital R	0.846	0.846	0.422	0.401	0.513	0.513	0.607	0.311	0.438
infraorbital L	0.607	0.607	0.819	0.846	0.311	0.483	0.846	0.513	0.483
Condylium R	0.846	0.311	0.291	0.513	0.311	0.03*	0.513	0.135	0.405
Condylium L	1.115	0.846	0.108	0.846	0.846	0.664	0.115	0.607	0.086
Gonion R	0.135	0.311	0.401	0.135	0.311	0.401	0.846	0.311	0.311
Gonion L	1.67	0.846	1	0.115	0.311	0.538	0.311	0.846	0.846
Mandibular Base R	0.846	0.513	0.293	0.223	0.311	0.311	0.011*	0.011*	0.008*
Mandibular Base L	0.311	0.513	0.223	0.135	0.223	0.607	0.009*	0.011*	0.032*
Pogonion	1	0.311	0.28	0.846	0.846	0.2	0.607	0.846	0.311
Menton	0.483	0.311	0.405	0.846	0.846	0.311	0.311	0.846	0.028*
Coronoid R	0.607	0.607	0.662	0.311	0.846	0.069*	0.846	0.513	0.651
Coronoid L	0.223	0.115	0.738	0.846	0.084	0.568	0.513	0.513	0.229

 A high ICC ( > 0.75%) was obtained by comparing the *x, y,* and *z* values at the location of landmark points. Excellent ICC > 0.75 was for 81.7% and poor < 0.40 was 7.5% in the interobserver evaluation. Data showed that 25 points had excellent concordance on the *x*-plane, 25 on the *y*-plane, and 26 on the *z*-plane (0.75%) (Table 5). One point had poor concordance on the* x*-plane condylium R, 4 on the *y*-plane; nasion, the frontomalar suture R, gonion L and menton, and 2 on the *z*-plane, menton and pogonion. Differences in millimeters for points of poor concordance ranged from 0.3–5 mm (Table 6).

**Table 5:** Evaluation of interobserver concordance with intraclass correlation analysis

**Coordinates**								
**Range **	**x**		**y**		**z**		**Total **	
	**n**	**%**	**n**	**%**	**n**	**%**	**n**	**%**
0.75-1 Excellent	25	80.6	25	80.6	26	83.9	76	81.7
0.60 – 0.74 Good	3	9.7	0	0.0	0	0.0	3	3.2
0.40-0.59 Sufficient	2	6.5	2	6.5	3	9.7	7	7.5
< 0.40 Poor	1	3.2	4	12.9	2	6.5	7	7.5
Total	31	100	31	100	31	100	93	100

**Table 6:** The difference in mm of the reference points with poor concordance

	**N_Y**	**Right fronto-malar suture_y**	**CondyliumR_x**	**Gonion L_y**	**Pogonion_z**	**Menton_y**	**Menton_z**
Total avg (mm)	0.3	2.0	5.0	2.6	2.4	0.8	0.4
Total SD	0.3	0.3	8.6	1.2	3.0	0.2	0.2

## Discussion

 Previous studies that dealt with the evaluation of the reliability and reproducibility of 3D reference points, used their anatomical definitions to locate them in the CBC.^5,14-16^ In this study, a detailed description was made of how to locate the reference points in each of the planes of space, as suggested by de Oliveira et al.^17^ We show the sequence in which the cutting planes must be modified, and we mention the structures to take into account the location of the landmark point, thus creating a universal concept to achieve the marking of the point consistently.

 In this study, 31 reference points were marked, most of them described in the literature,^18^ except for the mandibular right and left base points, Mx3 right and left and Mx6, right and left. These reference points were described for the first time in this study. Since variations have been found due to the poor description of the reference points.^17,19,20^ Here, the reference points were described three dimensionally on rigid structures to make their marking easier, modifying the way of locating the sella turcica.

 The results indicate that repeatability and concordance are achieved by marking the reference points proposed in this research. In a systematic review on the reliability of marking points in CBCT, the differences in a cephalometric analysis became clinically significant when the difference was greater than 0.5 mm.^3^

 In the intraobserver analysis of this study, the points did not present significant variations in more than one plane, except the right and left mandibular base point, which varied in the three planes of space. This point was described for the first time in this study and the description and understanding may be complex to achieve a good degree of precision. In the evaluation of interobserver concordance, a high correlation was found in 81.7% of points, a good correlation in 3%, a sufficient 7%, and a poor concordance in 7%. In this study, as well as in other studies where the repeatability and reliability of new reference points have been evaluated, it has been found that more than 80% of the points show high concordance.^13,21,22^

 In the points that showed poor ICC, there was variation in one of the three planes, except for the menton point, with poor concordance in the *y* and *z* planes. On the *y* and *z* planes, there points were cataloged with poor or high correlation, showing the thin line between concordance and not. On the *z*-plane, the points with high concordance were marked more frequently. The highest frequency of poor reliability was on the *y* plane as indicated by de Oliveira et al.^17^

 Nasion has been reported in the literature as one of the least variable points, since being on the midsagittal plane, it has been routinely visualized in the 2D view, which makes it easier to understand and locate,^23^ this is consistent with other studies where it turns out to have high reliability ICC (*x*: 0.87; *y*: 0.98; *z*: 0.97)^17^ (*x*: 0.98; *y*: 1; *z*: 0.98).^20^ In contrast, in our study, nasion was found to be located with more difficulty in the *y*-plane (ICC = 0.07), This may be due to the difficulty in finding the deepest point in the concavity of the floor of the sella turcica.

 On the *x*-plane for condylium R, while in other studies it was located with high concordance (ICC > 0.75) (*x*: 0.97), In the study by Chien et al^12^ bilateral evaluation is not reported they speak only of a condylium point while in this study we evaluated right and left condylium; they also omitted the *z*-plane to make the comparison with the 2D image. In Oliveira and colleagues’ study, left and right condylium are evaluated and the ICC for condylium R on the *x*-plane(0.46) shows sufficient concordance but is closer to our finding; on the *y*-plane, the data of high concordance coincide, and differs again on the *z*-plane(ICC = 0.28), which contrasts with the *z*-plane of our study (ICC = 0.99),^17^ the poor concordance of condylium R on the *x*-plane was also found in the study by Neiva et al^13^ (ICC = 0.21).

 The point located on the frontomalar suture R is evaluated in a few studies; in Neiva et al^13^ poor concordances were found on the *x-*plane and high on *y,z*; while in this study poor concordance was found on the *y*-plane and high in *x*,*z* and de Oliveira et al^17^ found that the point is concordant in *x,y,z*.Other points with poor concordance in this study were menton and pogonion, results that radically contrast with the results of high concordance found in the literature.^12,13,16,17^

 Gonion is one of the points reported with high marking difficulty, but in this study, it turned out to be a reliable point, perhaps due to the description that was made in the three planes of space using reference structures such as the mandibular ramus and base.^24^ However, the error associated with the gonion point is in the vertical plane as seen with the ICC of Gonion L on the *y*-plane (0.32) which may favor the presence of errors in measurements on the vertical plane having gonion as reference. The fact that there are three coordinates for the identification of a reference point adds to the risk of variability in the repeatability of the data. Therefore, the selection of the segment for marking the reference point must be carefully guided by the structures to be evaluated and properly managed by the visualization tools.

 Points with poor concordance in this study do not follow a pattern when compared to other studies. Generally, one of the planes shows poor concordance, while the remaining two show sufficient, good, or excellent. In this evaluation, discrepancies are observed in different planes, and two points were observed, nasion and menton with poor concordance in the two planes. It has already been mentioned that it is impossible to locate a point of reference according to its definition without a margin of error, however, all efforts are aimed at minimizing non-concordance.^25^

 In the points where poor concordance was found, the average difference in millimeters varies from 0.3 mm to 5 mm. The greatest discrepancy in this study is reported for condilium R on the *x*-plane with an average difference of 5 mm, other authors such as Lagravère et al^26^ also report variations greater than 1 mm.

 With the frequent variability that can be found on the *y*-plane and for points that are located on convex surfaces such as the Gonion point, an average difference is reported for Gonion L in *y* of 2.6 mm, while previously it had been reported greater difference (5.5 mm).^26^ Nasion point, in this study, had poor concordance in the *y*-plane and with an average difference of 0.3 mm, being found in the points with smaller difference averages.

 When analyzing the repeatability of the intraobserver marking pattern, suggests that with a complete description of the reference points, a clear training, a person with basic knowledge of image analysis and software management can locate the reference points properly.

 It has been shown that the location of the reference points in a CBCT is carried out in a reliable and repeatable way,^3^ however, due to the variations that occur in the manual location of the reference points, studies have been carried out that claim that one of the multiple functions of 3D craniofacial analysis is the automatic identification of points in the CBCT image.^27^ No consensus has been reached regarding the description of reference points for three-dimensional cephalometric analysis, therefore, the next step in this line of research will be to apply a three-dimensional analysis as a diagnostic tool. With the incursion of artificial intelligence tools and deep learning,^28^ an additional panorama opens for segmentation techniques and automatic identification of craniofacial structures, which will further facilitate this field of intuitive diagnosis.

## Conclusion

 Intraobserver concordance analysis indicated that the location of anatomical reference points on bimaxillary CBCT is performed with great reproducibility by interpreting their location with a clear description in the three planes of space. There are still important reference points for morphological analysis, that because they are located in convex areas with variable bone densities, are difficult to standardize. Variability in the manual marking of reference points may be determined by different visualization factors and clinical experience. The systematized location of the reference points would contribute to the reduction of such variability.

## Competing Interests

 The authors declare that there is no conflict of interest regarding the publication of this article.

## Ethical Approval

 This study was approved by the ethics committee of the Universidad del Valle under the act number 234-019.

## Funding

 This research did not receive any specific grant from funding agencies in the public, commercial, or not-for-profit sectors.
